# Effects of a postnatal *Atrx* conditional knockout in neurons on autism-like behaviours in male and female mice

**DOI:** 10.1186/s11689-020-09319-0

**Published:** 2020-06-24

**Authors:** Nicole Martin-Kenny, Nathalie G. Bérubé

**Affiliations:** 1 Department of Paediatrics, Schulich School of Medicine and Dentistry, Western University, London, Ontario Canada; 2 Department of Anatomy and Cell Biology, Schulich School of Medicine and Dentistry, Western University, London, Ontario Canada; 3 Division of Genetics and Development, Children’s Health Research Institute, London, Ontario Canada; 4 Department of Oncology, Schulich School of Medicine and Dentistry, Western University, London, Ontario Canada

**Keywords:** Autism spectrum disorder, ATRX, Social behaviours, Repetitive behaviours, Startle response, Genetically engineered mice, Cre/loxP system

## Abstract

**Background:**

Alpha-thalassemia/mental retardation, X-linked, or *ATRX*, is an autism susceptibility gene that encodes a chromatin remodeler. Mutations of *ATRX* result in the ATR-X intellectual disability syndrome and have been identified in autism spectrum disorder (ASD) patients. The mechanisms by which *ATRX* mutations lead to autism and autistic-like behaviours are not yet known. To address this question, we generated mice with postnatal *Atrx* inactivation in excitatory neurons of the forebrain and performed a battery of behavioural assays that assess autistic-like behaviours.

**Methods:**

Male and female mice with a postnatal conditional ablation of ATRX were generated using the Cre/lox system under the control of the *αCaMKII* gene promoter. These mice were tested in a battery of behavioural tests that assess autistic-like features. We utilized paradigms that measure social behaviour, repetitive, and stereotyped behaviours, as well as sensory gating. Statistics were calculated by two-way repeated measures ANOVA with Sidak’s multiple comparison test or unpaired Student’s *t* tests as indicated.

**Results:**

The behaviour tests revealed no significant differences between *Atrx*-cKO and control mice. We identified sexually dimorphic changes in odor habituation and discrimination; however, these changes did not correlate with social deficits.

**Conclusion:**

The postnatal knockout of *Atrx* in forebrain excitatory neurons does not lead to autism-related behaviours in male or female mice.

## Background

Autism spectrum disorder (ASD) is a behaviourally defined condition characterized by deficits in social and communicative abilities, impaired sensory gating, as well as the presence of stereotyped behaviours [1, 2]. Recent work has highlighted the important contribution of *de novo* variants and inherited copy number variants in ASD, confirming a strong genetic component of this disease [1–3]. Numerous autism susceptibility genes have been identified and shown to share commonalities in synaptic, transcriptional, and epigenetic mechanisms [4–6]. Mouse models have typically been used to investigate the behavioural implications of genetic mutations associated with ASD [7, 8]. However, these studies often omit the investigation of the sex-specific effects of these genetic mutations, limiting the potential translational applications. In the general population, ASD occurs at a 4:1 male:female ratio, highlighting the need to study the outcome of genetic mutations in both male and female model systems [1, 2].

In this study, we describe the impact of targeted inactivation of *Atrx* in glutamatergic neurons on behaviours related to autism in male and female mice. ATRX belongs to the SWI/SNF family of chromatin remodeling factors [9, 10]. Mutations in the *ATRX* gene are associated with an intellectual disability syndrome referred to as ATR-Xsyndrome, characterized by autistic-like behaviours in addition to cognitive deficits, intellectual disabilities, and developmental delays [11]. Furthermore, autistic carriers of rare mutations in *ATRX* have been discovered and missense variants in *ATRX* have been identified in male ASD patients. Interestingly, female carriers of *ATRX* mutations experience skewed X-inactivation, and as a result, are asymptomatic [12–18].

Previous studies have demonstrated that the loss of *Atrx* in the mouse forebrain causes changes in gene expression [19, 20]. Specifically, transcription of autism susceptibility genes, including the monogenic *Neuroligin-4* (*Nlgn4)*, are altered upon loss of *Atrx* [19]. Additionally, sexually dimorphic transcript changes have been revealed in the adult mouse hippocampus upon the loss of *Atrx* in excitatory neurons [21]. However, autistic behaviours were not evaluated in that report and should be addressed given the link between *ATRX* mutations and ASD.

In this study, we characterize the impact of *Atrx* loss in neurons on autistic-like behaviours in male and female mice. We used the AtrxCamKIICre model [21] where *Atrx* is ablated in forebrain excitatory neurons postnatally, thus bypassing deleterious effects of ATRX loss-of-function previously observed in neural progenitors during brain development [22, 23]. An array of behavioural assays was performed to investigate the presence of autistic-like behaviours, including deficits in sociability, altered sensory gating, and the presence of repetitive or stereotyped behaviours. These investigations revealed minimal behavioural deficits related to autism in both male and female mice. Interestingly, we identified changes in olfaction, particularly odor discrimination, in both male and female mice upon the conditional loss of *Atrx* in neurons. Overall, this study demonstrates that a conditional loss of *Atrx* in forebrain excitatory neurons postnatally does not result in typical autistic-like traits in male or female mice.

## Materials and methods

### Animal care and husbandry

Mice were exposed to a 12-h light/12-h dark cycle and with water and chow ad libitum. The *Atrx*^loxP^ females (129/Sv background) have been described previously [23]. *Atrx*^loxP^ mice were mated with C57BL/6 mice expressing Cre recombinase under the control of the *αCaMKII* gene promoter [24]. The progeny includes hemizygous male mice that produce no ATRX protein in forebrain excitatory neurons (*Atrx*-cKO^MALE^). The *Atrx*-cKO males were mated to *Atrx*^loxP^ females to yield homozygous deletion of *Atrx* in female mice (*Atrx*-cKO^FEMALE^). Male and female littermate floxed mice lacking the Cre allele were used as controls (Ctrl^MALE^; Ctrl^FEMALE^). Consequently, male and female mice are from different hybrid generations. Control littermates from the same hybrid generation as the corresponding conditional knockout mice were used for behavioural assays. Genotyping of tail biopsies for the presence of the floxed and Cre alleles was performed as described previously [23]. Conditional loss of the ATRX protein in postnatal neurons was previously verified in adult *Atrx*-cKO^MALE^ and *Atrx*-cKO^FEMALE^ forebrain [21]. All procedures involving animals were conducted in accordance with the regulations of the Animals for Research Act of the province of Ontario and approved by the University of Western Ontario Animal Care and Use Committee (2017-048). Behavioural assessments started with less demanding tasks and moved to more demanding tasks in the following order: open-field test, marble-burying assay, induced self-grooming, pre-pulse inhibition (PPI) and startle response, social approach, and 3-chamber social tests. ARRIVE guidelines were followed: mouse groups were randomized, experimenters were blind to the genotypes, and software-based analysis was used to score mouse performance in all the tasks. All behavioural tasks were performed between 9:00 AM and 4:00 PM. All behavioural assays were performed when mice were between 3 and 7 months of age. Three cohorts of male and female mice were used to reach the final sample size (Ctrl^MALE^: 17; *Atrx*-cKO^MALE^: 10; Ctrl^FEMALE^: 13; *Atrx*-cKO^FEMALE^: 13). Statistics were calculated by two-way repeated measures ANOVA with Sidak’s multiple comparison test or unpaired Student’s *t* tests, as indicated in the figure legends.

### Odor habituation and discrimination

The odor habituation and discrimination assay was performed as previously described [25] to assess olfaction. Individual mice were placed into a clean cage with a wire lid and allowed to habituate to the testing room for 30 min. The mice were then presented with an odor on a cotton swab (either almond, banana, or water as a control) for a 2-min trial. For each trial, 50 μl of water, almond extract, or banana extract (club house) was pipetted onto the tip of a cotton swab and the swab was then secured to the wire cage top through the water bottle opening. The mice were presented with the same odor three times before being presented with a new odour, for a total of nine trials. During the 2-min trials, the amount of time that the mouse spent sniffing the odor was recorded by an investigator blind to the genotype. Sniffing was defined as the animal’s nose being in proximity to the cotton swab (2 cm or closer), and oriented toward the swab.

### Social approach

This test was performed as previously described [26] to assess for sociability with conspecific mice. For two consecutive days prior to the test day, individual mice were habituated to the open area for 10 min. On the test day, pairs of unfamiliar, same-sex conspecific mice were placed into the cage. Behaviour of the mice was recorded by the AnyMaze software and video-tracking system. The time spent in social interaction, defined as the experimental mouse sniffing the stranger mouse, was manually scored by investigators unaware of the genotype.

### Three-chamber social tests (social preference and novelty)

The social preference and social novelty assessments were performed as described [26, 27] with minor modifications. Individual mice were placed in the 3-chambered box and allowed to freely explore the arena during a 10-min habituation period. After the habituation period, an unfamiliar, same-sex mouse of a different genotype (stranger 1) was placed in one of the side chambers under a wire cage. An identical wire cage containing an inanimate object was placed in the opposite chamber. The test mouse was then allowed to explore the entire 3-chambered arena for 10 min. The amount of time spent in each chamber was recorded by the AnyMaze video-tracking system. Following this period, a second unfamiliar, same-sex mouse of a different genotype (stranger 2) was placed into the wire cage previously containing the inanimate object. The test mouse was then allowed to explore the 3-chambered arena for 10 min. The amount of time spent in each chamber was recorded by the AnyMaze video-tracking system. Based on the amount of time spent in each chamber, a ‘sociability index’ and a ‘social novelty index’ was calculated as previously described [27]. The sociability index was calculated as time_stranger_/(time_stranger_ + time_object_) × 100. The social novelty index was calculated as time_novel_/(time_novel_ + time_familiar_) × 100.

### Marble burying

The test was performed as previously described [28] with modifications to evaluate repetitive digging behaviour. Mice were brought into the test room to habituate in their home cages for approximately 30 min prior to the test. The test cages were filled with 4 cm of wood-chip bedding, with 12 evenly spaced glass marbles placed on the surface. Individual mice were then placed in the test cage and permitted to explore for 30 min. Following the test, the number of marbles buried (> 3/4 surface covered) was counted and recorded by investigators blind to the genotype.

### Induced self-grooming

The test was performed as previously described [27, 29] to evaluate repetitive grooming tendencies. Mice were individually habituated in an empty test cage for 30 min prior to the test. To amplify natural grooming tendencies, mice were misted with water 3 times at 10 cm distance of the upper-back. Following this misting, the grooming behaviour of each mouse was recorded by the Anymaze video-tracking system for 30 min. The time that each individual mouse spent grooming during this 30-min trial was manually scored by the rater, unaware of the genotype.

### Open-field test

Mice were brought into the testing room to habituate in their home cages approximately 30 min prior to the test. Mice were placed in a 20 cm × 20 cm arena with 30 cm high walls. Locomotor activity was automatically recorded in 5-min intervals over 2 h (AccuScanInstrument) [30]. For each mouse the number of vertical episodes was assessed.

### Pre-pulse inhibition of the startle response

The pre-pulse inhibition and startle response tests were performed as previously described [31] to assess sensory gating. Mice underwent two days of habituation prior to the testing day, to acclimate the mice to the apparatus. During this habituation, mice were individually placed in the chamber apparatus and exposed to background noise (65 db) for 5 min (SR-LAB, San Diego Instruments). On the test day, individual mice were placed in the chamber and acclimated for 10 min with background noise. The mice then underwent a habituation block, consisting of 50 acoustic startle trials, with 20 ms stimulus of 115 db, and intertrial interval of 20 s. After the habituation block, mice underwent a prepulse-inhibition block consisting of ten sets of five types of trials randomly ordered with variable intertrial intervals of 10, 15, or 20 s. Four of the five trial types consisted of prepulses (intensity of 75 or 80 db, length of 20 ms), separated from the startle stimulus (intensity of 115 db, length of 40 ms) by an interstimulus interval of either 30 ms or 100 ms. The fifth trial type was a startle pulse alone. The startle response was measured by the movement of the mouse on the platform, which generates a transient force analyzed by the software. The startle magnitude recorded was an average for the ten trials of each trial type and startle magnitudes of pre-pulse trials were normalized to the pulse-only trial.

## Results

### Sexually dimorphic olfaction differences in Atrx-cKO mice

**Fig. 1:**
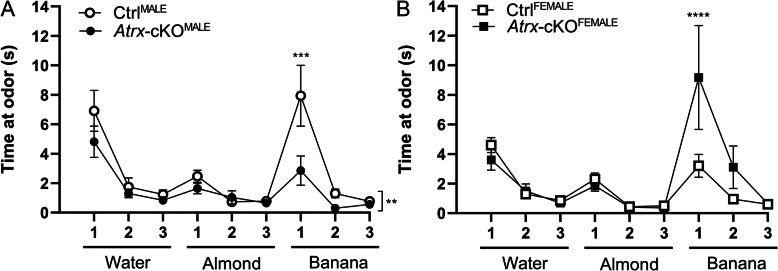
*Atrx*-cKO^MALE^ and *Atrx*-cKO^FEMALE^ mice exhibit differences in olfaction during the odor habituation and discrimination assay. The amount of time spent sniffing a cotton swab saturated with an odor during nine, 2-min trials. **a***Atrx*-cKO^MALE^ (*n* = 24) mice spend less time sniffing cotton swabs with corresponding odors compared to Ctrl^MALE^ (*n* = 17) (twANOVA, *F*_(1,351)_ = 8.203, **p = 0.004; mc, ***p < 0.001). **b***Atrx*-cKO^FEMALE^ (*n* = 19) mice spend more time sniffing the cotton swab with the banana odor when first exposed to the scent compared to Ctrl^FEMALE^ (*n* = 15) (twANOVA, *F*_(1,288)_ = 3.188, *p* = 0.075; mc, ****p < 0.0001). Error bars: ±SEM

As olfactory impairments can confound the interpretation of other tests, especially social behaviour assays, we first wanted to address whether the loss of *Atrx* in excitatory neurons of the forebrain alters olfaction in male and female mice. To do this, we performed the odor discrimination and habituation assay [25]. In this test, mice were presented with multiple odors for 2-min trials, during which the amount of time spent sniffing the odor was recorded. During this test, *Atrx-*cKO^MALE^ mice spent significantly less time sniffing the odors throughout the nine trials compared to Ctrl^MALE^ mice (ANOVA, **p = 0.004; Fig. 1a). In particular, *Atrx-*cKO^MALE^ mice spent significantly less time sniffing the cotton swab when first presented with the banana odor (multiple comparisons, ***p < 0.001). There was no significant difference in the overall amount of time spent sniffing the odors throughout the test between *Atrx-*cKO^FEMALE^ and Ctrl^FEMALE^ mice. However, *Atrx-*cKO^FEMALE^ mice did spend significantly more time sniffing the cotton swab when first presented with the banana odor (multiple comparison, ****p < 0.0001; Fig. 1b). Overall, the results of this test suggest that the loss of *Atrx* in forebrain excitatory neurons results in sexually dimorphic changes in olfaction that must be considered in subsequent behaviour testing of these mice.

### Social assays reveal no deficits in Atrx-cKO^MALE^ and Atrx-cKO^FEMALE^ mice

**Fig. 2:**
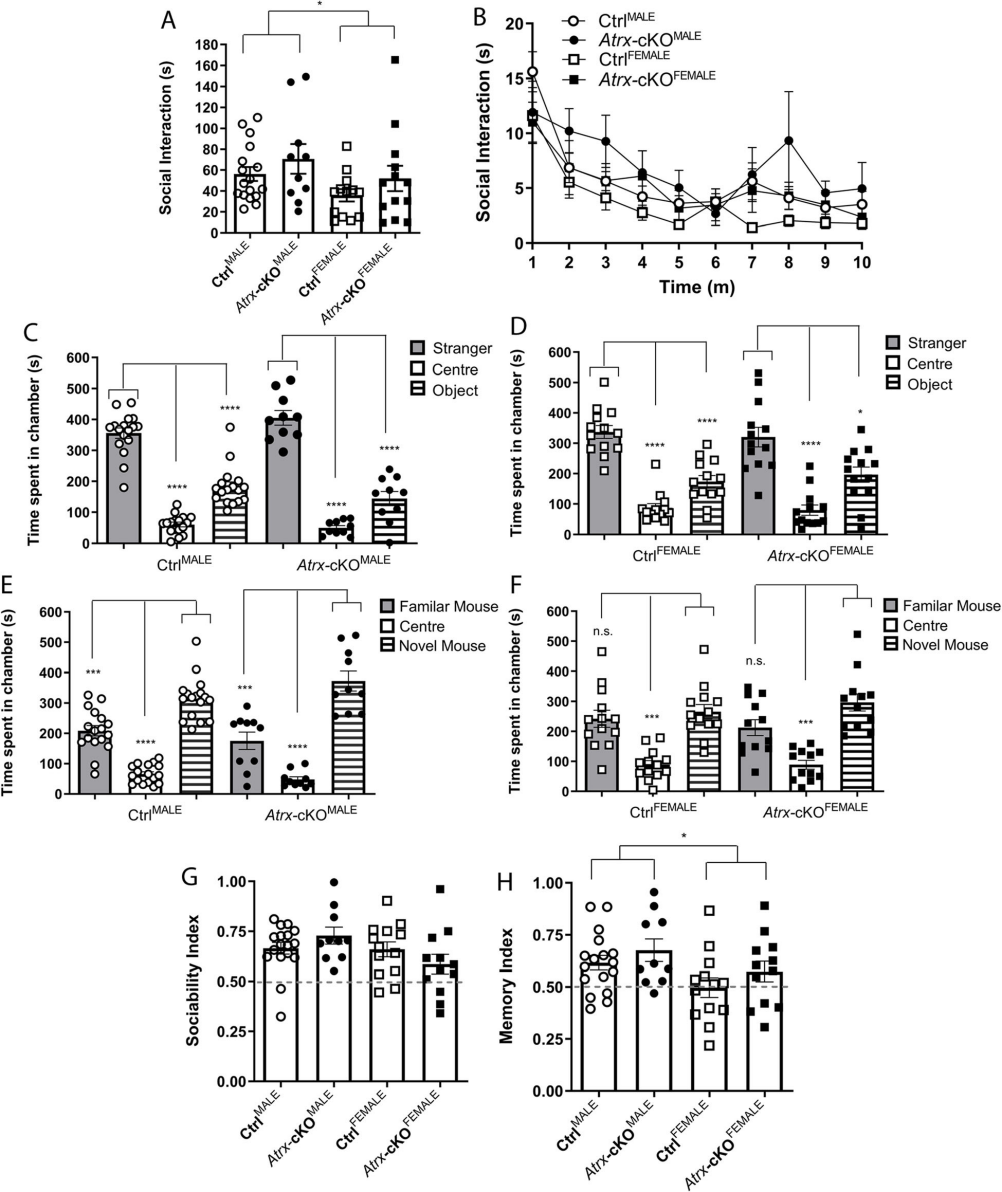
Social behaviour assays reveal lack of genotypic difference between *Atrx*-cKO and control mice. **a** Total amount of social interaction with a mouse conspecific for genotype and sex during the 10-min social approach assay (twANOVA, genotype, *F*_(1, 49)_ = 2.496, *p* = 0.121; twANOVA, sex, *F*_(1, 49)_ = 4.086, *p = 0.048). **b** Social interaction over 1-min intervals during the social approach assay (twANOVA, genotype, *F*_(1, 51)_ = 1.489, *p* = 0.228; twANOVA, sex, *F*_(1, 51)_ = 3.425, *p* = 0.070). **c** Amount of time Ctrl^MALE^ (twANOVA, *F*_(2, 32)_ = 78.39, ****p < 0.0001; mc, ****p < 0.0001) and *Atrx*-cKO^MALE^ (twANOVA, *F*_(2, 18)_ = 58.26, ****p < 0.0001; mc, ****p < 0.0001) spent in the empty centre chamber and chambers containing either a stranger mouse or novel object in the social preference assay. **d** Amount of time Ctrl^FEMALE^ (twANOVA, *F*_(2, 24)_ = 31.39, ****p < 0.0001; mc, ****p < 0.0001) and *Atrx*-cKO^FEMALE^ (twANOVA, *F*_(2, 24)_ = 14.88, ****p < 0.0001; mc, *p < 0.05, ****p < 0.0001) spent in chambers containing either a stranger mouse or novel object, or the empty centre chamber **e**. Time spent in the empty centre chamber and chambers containing a familiar mouse or a novel mouse in the social novelty assay for **e**. Ctrl^MALE^ (twANOVA, *F*_(2, 32)_ = 51.38, ****p < 0.0001; mc, ***p < 0.001, ****p < 0.0001) and *Atrx*-cKO^MALE^ mice (twANOVA, *F*_(2, 18)_ = 27.26, ****p < 0.0001; mc, ***p < 0.001, ****p < 0.0001), and **f** Ctrl^FEMALE^ (twANOVA, *F*_(2, 24)_ = 11.77, ***p < 0.001; mc, ***p < 0.001) and *Atrx*-cKO^FEMALE^ mice (twANOVA, *F*_(2, 22)_ = 12.50, ***p < 0.001; mc, ***p < 0.001). **g** The sociability index for each mouse was calculated as the time spent in the stranger mouse chamber, divided by the total time in the stranger mouse and novel object chambers (twANOVA, genotype, *F*_(1, 28)_ = 0.019, *p* = 0.890; twANOVA, sex, *F*_(1, 48)_ = 3.574, *p* = 0.065). **h** The social memory index for each mouse was calculated as the time spent in the novel mouse chamber, divided by the total time in the familiar mouse and novel mouse chambers (twANOVA, genotype, *F*_(1, 48)_ = 2.217, *p* = 0.143; twANOVA, sex, *F*_(1, 48)_ = 5.811, *p = 0.019). *Atrx*-cKO^MALE^: *n* = 10, Ctrl^MALE^: *n* = 17, *Atrx*-cKO^FEMALE^: *n* = 13, Ctrl^FEMALE^: *n* = 13. Error bars: ±SEM

Given that *ATRX* mutations are associated with autistic traits in humans, we next sought to investigate if the loss of *Atrx* in forebrain excitatory neurons has an effect on social behaviour. Changes in sociability and social preference are some of the most common deficits observed in mouse models with autism-associated genetic mutations [26, 27, 32–34]. As such, we first investigated sociability of *Atrx-*cKO^MALE^ and *Atrx-*cKO^FEMALE^ mice by means of the social approach assay, as described previously [26]. There was no significant difference in the total amount of time that *Atrx-*cKO^MALE^ and *Atrx-*cKO^FEMALE^ mice spent interacting with a stranger mouse compared to controls (Fig. 2a). However, when these results were grouped and analyzed by sex, male mice (*Atrx-*cKO^MALE^ and Ctrl^MALE^) spent more time socially interacting with the stranger mouse compared to female mice (*Atrx-*cKO^FEMALE^ and Ctrl^FEMALE^) (ANOVA, *p = 0.048; Fig. 2a). When social interaction was analyzed over 1-min intervals during the 10-min test, there were no genotypic or sex-differences (Fig. 2b).

We also investigated social preference and social novelty in the three-chambered paradigm [27]. During the first part of the paradigm, social preference was assessed as mice were placed into a three-chambered apparatus and were free to explore between the chambers. The outer two chambers contained either a novel object or a stranger mouse, while the centre chamber remained empty. Both Ctrl^MALE^ and *Atrx-*cKO^MALE^ mice demonstrated a preference for the chamber containing a stranger mouse compared to the object and the empty chamber (multiple comparisons, ****p < 0.0001; Fig. 2c). Similarly, both Ctrl^FEMALE^ and *Atrx-*cKO^FEMALE^ mice preferred exploration of the stranger mouse (multiple comparisons, *p = 0.030, ****p < 0.0001; Fig. 2d). No genotypic differences were observed in social preference between groups (Fig. 2c, d).

Social novelty was investigated during the second part of the paradigm in which the outer chambers contained either the stranger mouse from the first part of the test (familiar mouse) or a novel mouse. Ctrl^MALE^ and *Atrx-*cKO^MALE^ mice both spent more time in the chamber containing the novel mouse compared to the familiar mouse and the empty chamber (multiple comparisons, ***p < 0.001, ****p < 0.0001; Fig. 2e). Interestingly, although Ctrl^FEMALE^ and *Atrx-*cKO^FEMALE^ mice both spent significantly less time in the empty chamber, neither demonstrated a preference for the novel mouse over the familiar mouse (multiple comparisons, ***p < 0.001; Fig. 2f). Sociability and social memory indexes were calculated based on the social preference and social novelty results. *Atrx-*cKO and Ctrl mice did not display genotypic or sex-differences in their sociability indexes (Fig. 2g). Similarly, there was no genotypic difference in social memory indexes, however, male mice (Ctrl^MALE^ and *Atrx-*cKO^MALE^) displayed a greater social memory index than female mice (Ctrl^FEMALE^ and *Atrx-*cKO^FEMALE^) (ANOVA, *p = 0.019; Fig. 2h). Altogether, these results demonstrate that the loss of *Atrx* in forebrain excitatory neurons postnatally does not result in social deficits in male and female mice.

### Repetitive behaviours are not altered in Atrx-cKO^MALE^ and Atrx-cKO^FEMALE^ mice

**Fig. 3:**
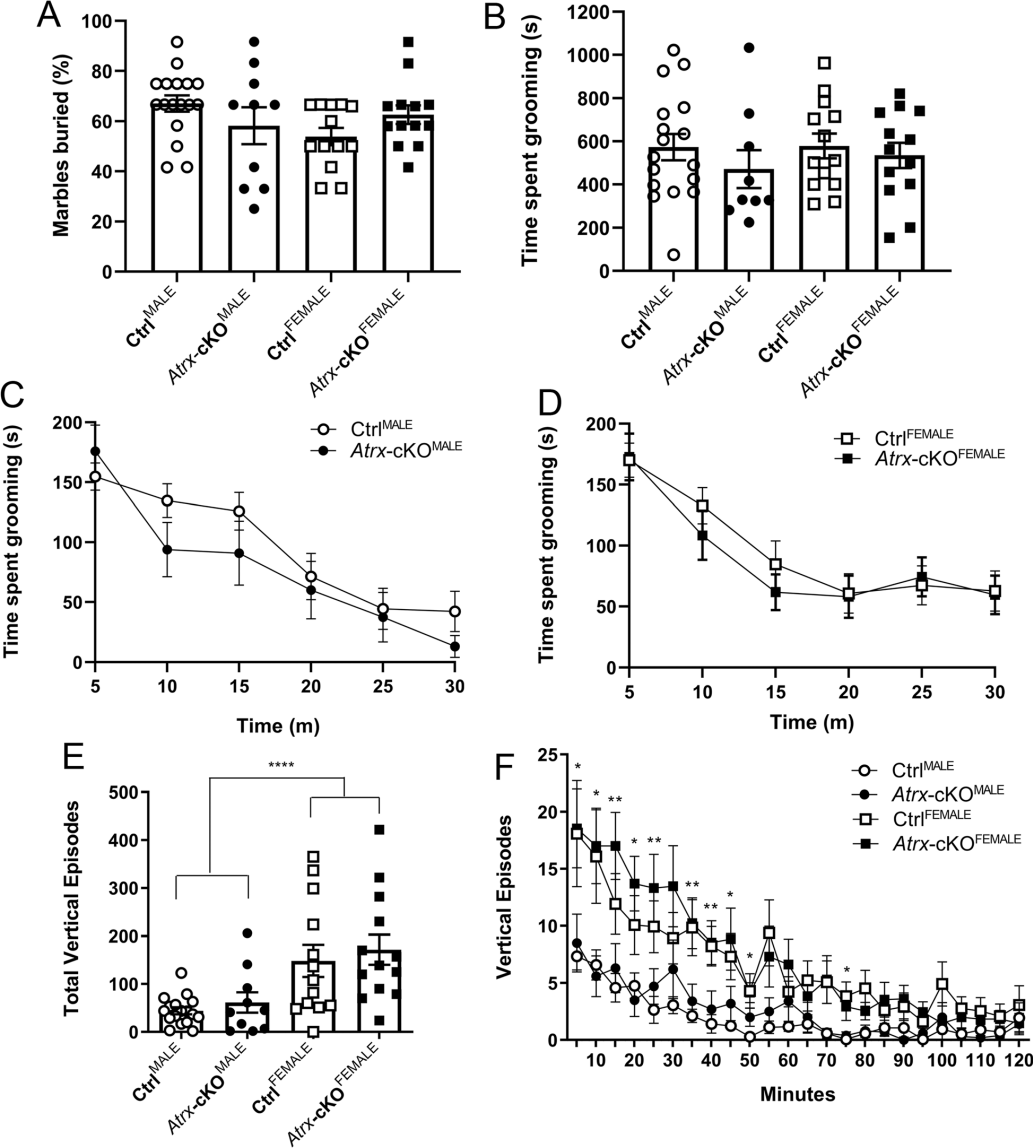
*Atrx*-cKO^MALE^ and *Atrx*-cKO^FEMALE^ mice do not display stereotyped behaviours. **a** Percentage of marbles buried during 30-min marble burying task (twANOVA, genotype, *F*_(1, 49)_ = 0.0003, *p* = 0.985; twANOVA, sex, *F*_(1, 49)_ = 1.038, *p* = 0.313; twANOVA, interaction, *F*_(1, 49)_ = 4.132, *p = 0.047). **b** Total time grooming during 30-min water-induced grooming task (twANOVA, genotype, *F*_(1, 48)_ = 1.200, *p* = 0.279; twANOVA, sex, *F*_(1, 48)_ = 0.268, *p* = 0.607). **c**, **d** Amount of time spent grooming over 5-min intervals during water-induced grooming task (twANOVA, males, *F*_(1, 44)_ = 2.386, *p* = 0.125; twANOVA, females, *F*_(1, 44)_ = 0.562, *p* = 0.455). **e** Total number of vertical episodes during 120-min open-field test (twANOVA, genotype, *F*_(1, 49)_ = 0.673, *p* = 0.416; twANOVA, sex, *F*_(1, 49)_ = 18.71, ****p < 0.0001). **f** Number of vertical episodes over 10-min intervals during the 120-min open-field test (twANOVA, genotype, *F*_(1, 51)_ = 0.866, *p* = 0.356; twANOVA, sex, *F*_(1, 51)_ = 19.19, ****p < 0.0001; mc, *p < 0.05, **p < 0.01). *Atrx*-cKO^MALE^: *n* = 10, Ctrl^MALE^: *n* = 17, *Atrx*-cKO^FEMALE^: *n* = 13, Ctrl^FEMALE^: *n* = 13. Error bars: ±SEM

Previous studies using autism mouse models have demonstrated that the mutant mice often present with repetitive and stereotyped behaviours [27, 34–37]. We tested for the presence of these repetitive and stereotyped behaviours in both *Atrx-*cKO^MALE^ and *Atrx-*cKO^FEMALE^ mice using various tests. The marble-burying assay was used to assess repetitive burying and digging by placing mice in a cage with 12 marbles and recording how many marbles were buried following a 30-min period. Percentage of marbles buried during the marble-burying assay was not significantly different when comparing *Atrx-*cKO^MALE^ and *Atrx-*cKO^FEMALE^ mice to their respective controls. There also was no difference when comparing between genotypes or sexes. However, the interaction was significantly different between groups, suggesting the loss of *Atrx* in forebrain excitatory neurons has opposing effects on marble burying when comparing male and female mice (ANOVA, *p = 0.047; Fig. 3a).

We also investigated the presence of repetitive grooming tendencies by misting mice with water to induce grooming behaviours. The total amount of time spent grooming during the 30-min induced self-grooming assay was not significantly different between *Atrx-*cKO mice and controls, or between sexes (Fig. 3b). When the results of this test were analyzed over 5-min intervals, similarly, there was no difference in the amount of time spent grooming between *Atrx-*cKO^MALE^ and Ctrl^MALE^ mice (Fig. 3c) or *Atrx-*cKO^FEMALE^ and Ctrl^FEMALE^ mice (Fig. 3d). Interestingly, results from the open field test show a significant increase in the number of vertical episodes (including rearing and jumping) of female mice (*Atrx-*cKO^FEMALE^ and Ctrl^FEMALE^ mice) compared to male mice (*Atrx-*cKO^MALE^ and Ctrl^MALE^) (ANOVA, ****p < 0.0001; Fig. 3e). When these results were analyzed in 10-min intervals, it is apparent that these sex-differences in vertical episodes occurred primarily within the first 60 min of the open-field test (multiple comparisons; *p < 0.05, **p < 0.01; Fig. 3f). In addition to these sex-differences, there was no genotypic difference between the total vertical episodes or the vertical episodes over time. Similarly, there were no significant differences between *Atrx-*cKO mice and controls when analyzing vertical episodes. These behavioural analyses suggest that the loss of *Atrx* in forebrain excitatory neurons postnatally does not result in repetitive or stereotyped behaviours typically associated with autism.

### Atrx-cKO^MALE^ and Atrx-cKO^FEMALE^ mice display typical startle response to acoustic stimuli

**Fig. 4:**
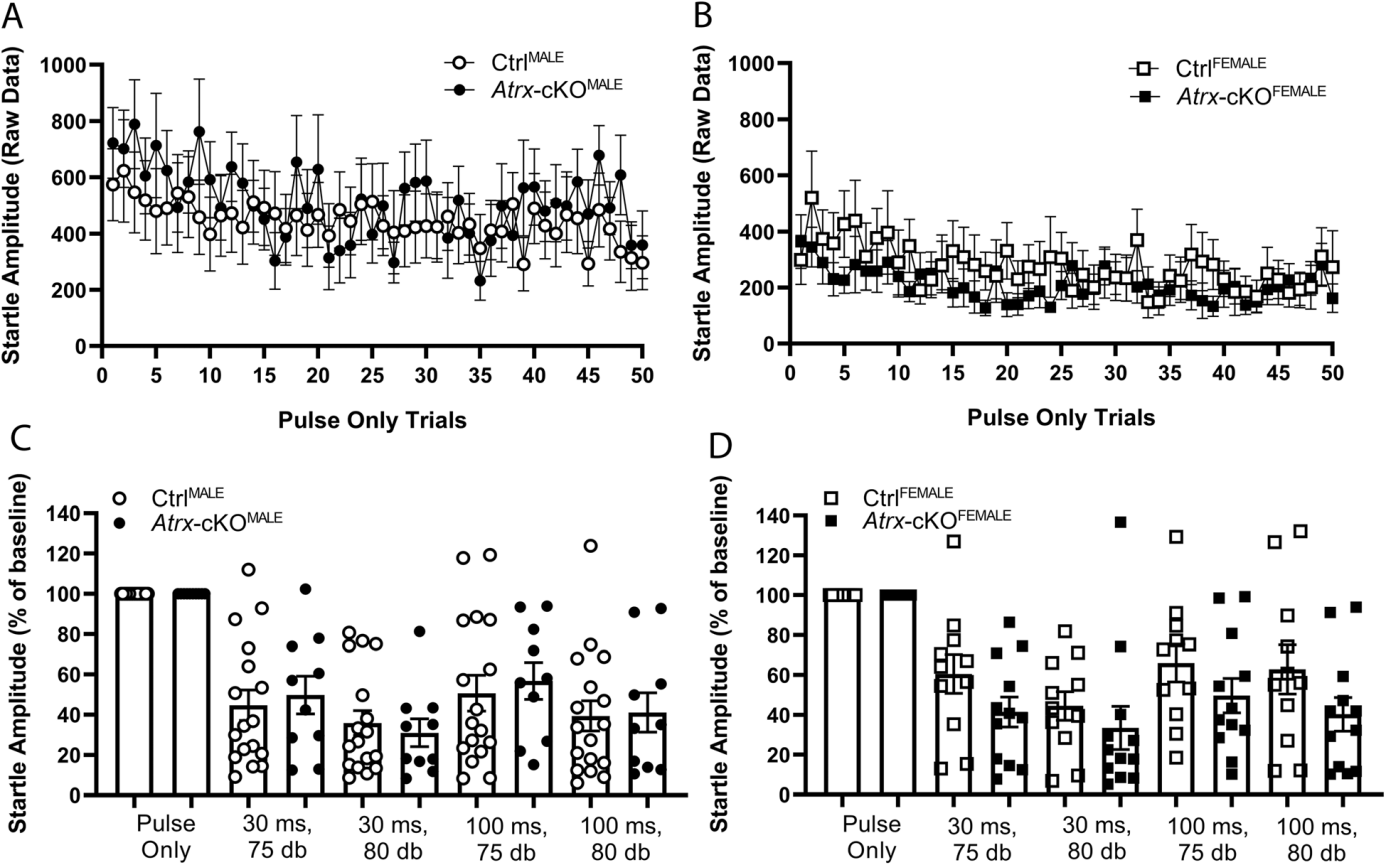
Acoustic startle response of *Atrx*-cKO mice is typical. **a**, **b** Quantification of startle responses (recorded in millivolts) to 50 “pulse only” trials (twANOVA, genotype, *F*_(1, 48)_ = 0.084, *p* = 0.773; twANOVA, sex, *F*_(1, 48)_ = 5.555, *p = 0.023). **c**, **d** Averaged pre-pulse inhibition for four trial types, varying in interstimulus intervals (30 ms or 100 ms) and pre-pulse intensity (75 db or 80 db). Startle responses for these trials are expressed as a percentage of the normalized “pulse only” trial (baseline) (Student’s *t* test, *Atrx*-cKO^MALE^, and Ctrl^MALE^ for each trial type, *p* > 0.05; Student’s *t* test, *Atrx*-cKO^FEMALE^ and Ctrl^FEMALE^ for each trial type, *p* > 0.05). *Atrx*-cKO^MALE^: *n* = 10, Ctrl^MALE^: *n* = 17, *Atrx*-cKO^FEMALE^: *n* = 11, Ctrl^FEMALE^: *n* = 12. Error bars: ±SEM

Previous studies have reported that rodent models with autism-associated genetic mutations can display an exaggerated startle response, or impaired pre-pulse inhibition, to an acoustic stimulus [38–41]. These impairments are associated with deficits in sensory gating and auditory processing often reported in ASD patients [42, 43]. As such, we wanted to investigate if *Atrx*-cKO mice display hypersensitivity to an acoustic startle stimulus. The pre-pulse inhibition and startle response assay using an acoustic stimulus was performed, as described previously [31]. Mice were placed in a chamber and exposed to 50 trials of an acoustic stimulus (20 ms, 115 db). *Atrx-*cKO^MALE^ and *Atrx-*cKO^FEMALE^ mice demonstrated a similar startle response to the acoustic stimuli compared to their respective controls (Fig. 4a, b). Additionally, there was no significant difference in startle responses when comparing genotypes (*Atrx-*cKO vs. Ctrl). Notably, there was a significant increase in the startle response of male mice (*Atrx-*cKO^MALE^ and Ctrl^MALE^) compared to female mice (*Atrx-*cKO^FEMALE^ and Ctrl^FEMALE^) (ANOVA, *p < 0.023). We also performed a set of trials that investigated pre-pulse inhibition to the acoustic stimulus by first exposing mice to a pre-pulse that preceded the acoustic stimulus. These trials varied in the intensity of the pre-pulse (75 db or 80 db), and the amount of time between the pre-pulse and the acoustic “pulse” stimulus (30 ms or 100 ms). Additionally, there was one trial that only involved a “pulse” without a pre-pulse. Startle responses to this “pulse-only” trial were used as a baseline, and results from all other trials were expressed as a percentage of this baseline. For all pre-pulse trials, both *Atrx-*cKO^MALE^ and *Atrx-*cKO^FEMALE^ mice did not demonstrate significant difference in their startle responses to the acoustic stimulus compared to the controls **(**Fig. 4c, d**).** Overall, these results suggest that the postnatal loss of *Atrx* in neurons does not result in an exaggerated startle response or impaired pre-pulse inhibition in male or in female mice.

## Discussion

In this study we present an assessment of the effects of a postnatal conditional knockout of the autism susceptibility gene *Atrx* on autistic-like behaviours in male and female mice. We provide evidence that the postnatal loss of *Atrx* in forebrain excitatory neurons does not result in social deficits, stereotypies and repetitive behaviours, or sensory gating deficits. We identified differences in olfaction for both male and female mice upon the postnatal conditional loss of *Atrx* in neurons; however, these differences in olfaction did not impair social behaviours.

Prior to investigating ASD-related behaviours, we first sought to determine whether *Atrx*-cKO adult mice present with any olfactory differences compared to controls. We performed the odor discrimination and habituation assay to assess if *Atrx*-cKO mice showed habituation to a repeatedly presented odor and were able to discriminate between a novel odor [25]. By establishing that experimental mice are able to detect and discriminate between odors, results of subsequent social behaviour assays can be more accurately interpreted. Results of the odor habituation and discrimination assay suggest that *Atrx*-cKO^MALE^ mice have deficits in olfaction, as they spent overall less time smelling multiple odors compared to controls. In particular, odor discrimination may be affected in *Atrx*-cKO^MALE^ mice as demonstrated by decreased time smelling a novel odor following repeated presentation of another. These differences in olfaction displayed by *Atrx*-cKO^MALE^ mice are important to consider when interpreting results from social behaviour assays that require odor discrimination. It is interesting to note that olfaction deficits have been reported in both ASD patients as well as models with autism-associated genetic mutations. In a recent clinical study, nine children with ASD demonstrated impaired olfactory adaption compared to a control group [44]. Additionally, another study reported that mice with haploinsufficiency of the autism-associated gene, T-box, Brain 1 (*Tbr1*), displayed impairments in olfactory discrimination [45]. Therefore, these impairments in olfaction, particularly odor discrimination, may be an indication of autistic-like features.

While there was no overall difference in olfaction when we compared *Atrx-*cKO^FEMALE^ mice to controls, *Atrx-*cKO^FEMALE^ mice demonstrated increased time spent sniffing the cotton swab when presented with the banana odor for the first trial. These results suggest that their odor discrimination may be heightened compared to that of Ctrl^FEMALE^. Altogether, although *Atrx*-cKO^MALE^ and *Atrx-*cKO^FEMALE^ mice display differences in olfaction compared to controls, we did not identify any genotypic-differences in social behaviours either in the social approach test or the 3-chamber paradigm. Therefore, any differences in olfaction of *Atrx-*cKO mice did not result in impairments in social recognition or discrimination based on olfactory cues.

Although there were no identified genotypic effects on the behaviours analyzed here, analysis of the data revealed potential sex-differences in sociability, social novelty, startle response, and anxiety levels. Sex differences in startle amplitude and marble burying have not yet been reported in the literature for the hybrid strain that was used in this study and should be repeated using a replication cohort. However, it is important to note that our experimental approach was not designed to detect differences based on sex, as male and female mice were obtained from different generations of strain hybrids.

The *Atrx*-cKO^FEMALE^ mice used in our study experience a complete loss of ATRX expression in forebrain excitatory neurons postnatally. However, in humans, females harbouring *ATRX* mutations are typically carriers and are asymptomatic due to skewed X-inactivation [12–18]. As such, the clinical relevance of any observed differences we observed in this model are limited. Nevertheless, our model allows the exploration of the basic biology of ATRX function in neurons and the potential effects on the behavioural outcomes. We theorize that the absence of autistic-like phenotypes observed in *Atrx*-cKO mice is due to the timing at which *Atrx* is deleted in forebrain excitatory neurons, which starts at 2–3 weeks of age. ASD is clinically defined as a developmental disorder due to the majority of symptoms becoming apparent in the first few years of life. Therefore, genetic mutations that contribute to autistic phenotypes may need to occur during embryogenesis or be inherited [1, 2, 4]. Future studies should utilize additional Cre/loxp systems to investigate if the loss of *Atrx* in differentiated forebrain excitatory neurons during embryogenesis leads to autistic-like behaviours in male and female mice.

## Conclusions

In conclusion, a postnatal conditional knockout of the autism susceptibility gene *Atrx* did not result in autistic-like behaviours in either male or female mice. Although changes in olfaction were observed in both male and female *Atrx-*cKO mice, these differences did not result in impaired social recognition or discrimination. These findings suggest that the postnatal loss of ATRX is insufficient to cause the subset of autistic behaviours tested here and support the idea that ASD is a developmental disorder where disruptions occur at early stages of brain development.

## Data Availability

All data generated or analysed during this study are included in this published article.

## Abbreviations

Atrx: Alpha-thalassemia mental retardation, X-linked
cKO: Conditional knockout
ASD: Autism spectrum disorder
twANOVA: Two-way ANOVA
mc: Multiple comparisons
